# Multi-Source Information Fusion for Environmental Perception of Intelligent Vehicles Using Sage-Husa Adaptive Extended Kalman Filtering

**DOI:** 10.3390/s25071986

**Published:** 2025-03-22

**Authors:** Yibo Meng, Huifang Kong, Tiankuo Liu

**Affiliations:** State Key Laboratory of High-Efficiency and High-Quality Conversion for Electric Power, Hefei University of Technology, Hefei 230002, China; mengyb1994@163.com (Y.M.); liutiankuo@mail.hfut.edu.cn (T.L.)

**Keywords:** intelligent driving, environmental perception, multi-source information fusion, Sage-Husa adaptive extended Kalman filtering, fading factor

## Abstract

With the rapid advancement of intelligent driving technology, multi-source information fusion has become a vital topic in the field of environmental perception. To address the fusion deviation resulting from changes in sensor performance due to environmental variations, this paper proposes a multi-source information fusion algorithm based on the improved Sage-Husa adaptive extended Kalman filtering (SHAEKF) algorithm. First, a multi-source information fusion system is constructed based on the vehicle kinematic model and the sensor measurement model. Then, the Sage-Husa adaptive fading extended Kalman filtering (SHAFEKF) algorithm is constructed by introducing a fading factor into the SHAEKF algorithm to enhance the influence of newly incoming data. Finally, the experimental results indicate that the positional average errors of the algorithm in the two scenarios are 0.137 and 0.071. When compared to the SHAEKF algorithm, the positional average errors have been reduced by 2.8% and 13.4%, while the mean squared errors have decreased by 64% and 72%. This demonstrates that the SHAFEKF algorithm offers high accuracy and low fluctuation, enhancing its adaptability in multi-source information fusion systems.

## 1. Introduction

With the rapid advancement of intelligent driving technologies for electric vehicles, environmental perception technology has increasingly gained traction. Currently, this field of research is recognized as a pivotal topic in the realm of intelligent driving [1]. Environmental perception encompasses the collection, processing, and analysis of information concerning both the driving environment and the vehicles themselves [2,3]. It serves as a vital foundation and prerequisite for the autonomous operation of smart electric vehicles. Consequently, enhancing the environmental awareness of intelligent driving systems is essential for improving their understanding of the vehicle’s operational context and its surroundings [4]. This complex research area has attracted considerable attention and sparked dynamic discussions among researchers worldwide. Environmental perception technology is advancing rapidly, and ongoing research and applications in this field will have significant and enduring implications [5].

Multi-source information fusion technology for environmental perception has emerged as a prominent research area among scholars [6]. The presence of numerous surrounding targets, high movement speeds, and significant randomness in driving environments often renders data collected from a single sensor inadequate to meet user requirements [7]. Consequently, researchers have begun investigating the fusion of multi-sensor detection data to provide accurate positional information on targets. The effective integration of detection data from multiple sensors has become a major focus in the fields of multi-sensor information fusion and environmental perception. This area has increasingly evolved into an interdisciplinary field that commands significant interest [8,9]. Unlike systems that depend on a single sensor, multi-sensor configurations leverage the complementary strengths of various sensors, achieving a higher level of data acquisition accuracy than any individual sensor can provide [10,11]. Moreover, the integration of multi-source sensor data across different layers can enhance the understanding of autonomous driving scenarios in intelligent vehicles, thus facilitating their efficient and stable operation within these environments [12].

A sensor fusion system represents an advanced information processing framework that facilitates real-time data collection from multiple sensors. These sensors possess both continuous and temporal characteristics, with interdependencies and influences among various types of information. Multi-source information fusion methods are critical to the domain of multi-sensor data fusion. Currently, these methods are classified into four primary categories: inference methods, classification methods, artificial intelligence methods, and filtering and estimation methods. Inference methods primarily rely on Dempster–Shafer (D-S) evidence theory, which effectively integrates information from multiple sensors using a distinctive evidence combination rule. This theory is extensively applied in scenarios involving uncertainty and ambiguity [13]. The widely utilized K-means algorithm in classification methods is effective for data clustering, as it groups similar samples, reduces data dimensionality, and eliminates outliers that deviate from the majority of data points [14]. Artificial intelligence methods predominantly rely on artificial neural networks for multisource information fusion, effectively integrating data from diverse sensors and extracting salient features [15]. By processing data from various sensors, the network learns the interrelationships among them, thereby enabling information fusion and optimization. Filtering and estimation methods have emerged as the most extensively studied and applied techniques due to their robustness and suitability for dynamic and complex environments, with the Kalman filter (KF) algorithm being a notable example [16].

The Kalman Filter (KF) algorithm is predicated on a linear dynamic model of the system [17]. By utilizing iterative state predictions and measurement updates, it recursively produces the optimal estimate of the system’s state. However, the traditional Kalman Filter is limited to linear systems, which has led researchers to create the Extended Kalman Filter (EKF) to address this constraint [18]. The EKF transforms nonlinear system equations into linear approximations through a first-order Taylor series expansion, using the current mean error and covariance. This approach effectively accommodates the nonlinear characteristics of the system. In contrast, the Unscented Kalman Filter (UKF) eliminates the need to linearize nonlinear systems [19]. It adopts a strategy that selects a set of deterministic points and employs weighted averaging to compute state estimates, thereby enhancing estimation accuracy. To tackle the issue of time-varying parameters in state estimation and prediction for dynamic systems, an adaptive Kalman filtering algorithm has been developed. This algorithm dynamically adjusts the filter parameters based on observational data to improve estimation accuracy. Among these techniques, Sage-Husa adaptive Kalman filtering notably enhances state estimation accuracy by dynamically modifying the noise covariance matrix within the Kalman Filter [20]. Zeyuan Luo et al. propose an AUKF based on a modified Sage-Husa filter and divergence calculation technique for multi-dimensional vehicle driving state observation. To mitigate the impact of transient disturbances on the subsequent process, the covariance matrix is updated upon detecting divergence [21]. Bai et al. propose an improved adaptive Unscented Kalman filter. This algorithm incorporates an adaptive mechanism that allows it to automatically adjust filtering parameters in response to environmental changes. As a result, it maintains a high level of positioning accuracy even in conditions of poor channel quality or significant variations in noise levels [22]. Dapeng Wang et al. proposed an adaptive robust UKF to address the multi-sensor fusion problem in nonlinear stochastic systems. Local filters were designed based on the covariance of redundant measurement noise isolated by state estimation, while Mahalanobis distance hypothesis testing theory was used to adaptively adjust the process noise covariance in real time, utilizing the innovation sequence and residual sequence [23].

Sensor performance is affected by variations in the driving environment, which can result in deviations in noise covariance. An inability to promptly and accurately estimate the measurement noise covariance may lead to inaccuracies in the residuals, subsequently diminishing the estimation accuracy in subsequent iterations. Liang Ma proposes an adaptive extended Kalman filter based on variational Bayesian and Sage-Husa prediction algorithms. This algorithm can adaptively modify the present motion model and uncertain measurement variance caused by stochastic ocean environmental noise [24]. Xinxin Yan proposes a novel Sage-Husa adaptive robust strong tracking Kalman filter by combining the Sage-Husa adaptive robust KF with the strong tracking KF. The modified Sage-Husa adaptive robust–STKF algorithm not only effectively filters random noise but also shows an excellent suppression of outliers [25]. Hongjian Jiao proposes enhanced modifications to the Sage-Husa Adaptive Kalman Filter (SHAKF) algorithm to address suboptimal observation weight distribution, introducing dual gross error detection and innovation-based adaptive correction of the forgetting factor in the iterative Kalman framework, thereby improving estimation accuracy and robustness [26].

This paper presents a multi-source information fusion system based on the Sage-Husa adaptive extended Kalman filtering algorithm. First, a sensor measurement model is developed based on the dynamic model of intelligent vehicles, leading to the establishment of a multi-sensor information fusion system. Then, a fading factor is incorporated into the Sage-Husa adaptive extended Kalman filtering algorithm, effectively reducing estimation errors while also mitigating the risk of filter divergence. Finally, a mobile robot is employed in a laboratory setting to simulate the perception scenarios of intelligent vehicles, enabling the verification of the fusion system’s accuracy and the effectiveness of the algorithms.

The major contributions of this paper are as follows:

1. A multi-sensor information fusion method is proposed to address the information offset issue of single sensors.
2. An improved SHAEKF algorithm is proposed, which introduces a fading factor to reduce estimation errors and suppress the possibility of filter divergence.
3. Experimental results indicate that the proposed method significantly improves obstacle detection accuracy, thereby enhancing the driving safety of intelligent vehicles.

The remainder of this paper is organized as follows. Section 2 establishes the dynamics model for electric vehicles. Section 3 proposes the multi-source information fusion system based on SHAFEKF, including the sensor system: SHAFEKF-based multi-source information fusion. Section 4 presents and analyzes the experimental results. Section 5 provides a summary of the paper and outlines directions for future research.

## 2. Electric Vehicle Dynamics Model

Intelligent vehicles signify a substantial enhancement in contemporary transportation technology. These vehicles combine multiple sensors, information processing algorithms, and dynamic motion models to facilitate autonomous navigation and decision-making within complex traffic environments, thus improving both safety and efficiency. The effective performance of intelligent driving vehicles depends on the precise modeling of their motion dynamics to accurately represent their operational status in real time within changing environments. In the examination of vehicle motion models, they are generally classified into two categories: first-order models and second-order models. First-order motion models, commonly known as linear motion models, primarily encompass the Constant Velocity (CV) model and the Constant Acceleration (CA) model. These linear models simplify the vehicle’s motion process by assuming that the vehicle travels exclusively in a straight line; however, they fail to adequately capture the complex dynamics associated with steering maneuvers. This simplification may result in inaccuracies regarding the vehicle’s actual motion performance, particularly in scenarios that necessitate steering control. In contrast, second-order motion models offer a more sophisticated and precise approach to describing vehicle motion. These models include the Constant Turn Rate and Velocity (CTRV) model and the Constant Turn Rate and Acceleration (CTRA) model. Compared to first-order models, second-order models comprehensively account for the motion characteristics of intelligent electric vehicles, particularly when their travel path necessitates steering. These vehicles often experience variations in measurement data and time intervals from different sensors, requiring motion models to be flexible enough to adapt to dynamic performance under varying conditions. In light of the aforementioned factors, this paper identifies the CTRV model as the most appropriate motion model for intelligent electric vehicles. The selection of this model not only provides a more accurate representation of the vehicle’s motion state but also establishes a theoretical foundation for implementing advanced intelligent driving systems, thereby enhancing adaptability and reliability in practical applications. This is crucial for advancing the deployment of intelligent electric vehicles in real-world road environments [27].

When the vehicle is in motion, the key real-time state variables related to its tracking target include

(1) $$g(t)=\left(x,y,v,\theta ,\omega \right)$$

where *x* and *y* are the target position, *v* is the target radial velocity, *θ* is the yaw angle, and ω is the yaw rate. Thus, the state transition function of the CTRV model is as follows:

(2) $$g(t+\Delta t)=\left[\begin{array}{c} \frac{v}{\omega }\sin(\omega \Delta t+\theta )-\frac{v}{\omega }\sin(\theta )+x(t)\\ -\frac{v}{\omega }\cos(\omega \Delta t+\theta )+\frac{v}{\omega }\cos(\theta )+y(t)\\ v\\ \omega \Delta t+\theta \\ \omega \end{array}\right]$$

Since this state transition function is nonlinear, it needs to be linearized, which involves calculating the corresponding Jacobian matrix. In the CTRV model, the partial derivatives of each element can be used to obtain the corresponding state Jacobian matrix.

(3) $$J=\left[\begin{array}{ccc} 1 & 0 & \frac{1}{\omega }(-\sin(\theta )+\sin(\Delta t\omega +\theta ))\\ 0 & 1 & \frac{v}{\omega }(-\cos(\theta )+\cos(\Delta t\omega +\theta ))\\ 0 & 0 & 1\\ 0 & 0 & 0\\ 0 & 0 & 0 \end{array}\right]$$

## 3. Multi-Source Information Fusion System

**Figure 1:**
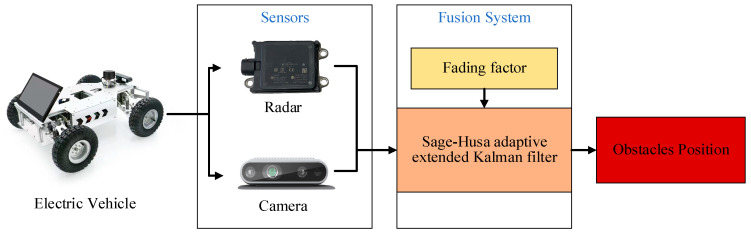
Schematic diagram of the fusion system.

This section describes a multi-source information fusion system grounded in the SHAEKF algorithm. It details the methods by which intelligent vehicles employ sensors, including radar and cameras, to gather and process data concerning surrounding obstacles. Following this, a fading factor is integrated to improve the SHAEKF algorithm, facilitating better fusion of the information obtained from both radar and cameras. The schematic representation of the proposed fusion system is depicted in Figure 1.

### 3.1. Sensor System

At present, the sensors used in environmental perception technology for autonomous driving mainly include cameras and millimeter-wave radar. Cameras deliver information that is both highly intuitive and detailed, closely mimicking human vision, allowing them to gauge the relative distance and velocity of objects in relation to the vehicle [28]. They present numerous benefits, such as an extensive monitoring range, significant data capacity, and cost efficiency. However, their effectiveness is notably compromised in conditions of low visibility, inadequate lighting, and reflective surfaces, making them less reliable in diverse weather situations. Millimeter-wave radar operates on the principles of the Doppler effect, and the data It gathers are expressed in polar coordinates. This form of technology offers advantages such as a compact design, broad bandwidth, substantial detection range, and robust anti-interference capabilities; nonetheless, it struggles to accurately detect target information within parallel planes. To improve the precision of target localization and enhance the overall robustness of the system, it is common practice to integrate multiple sensors for environmental perception.

The measurement equation for the camera Is expressed as follows:

(4) $$Z_{k}=\left[x,y,v\right]$$

where *x* and *y* are the target position and *v* is the target radial velocity.

The measurement equation for the millimeter-wave radar is expressed as follows:

(5) $$Z_{k}=\left[\rho ,\theta ,\rho^{\prime }\right]$$

where *p* represents the distance to the target in polar coordinates, *θ* represents the direction angle, and *p*′ represents the rate of change of the distance.

**Figure 2:**
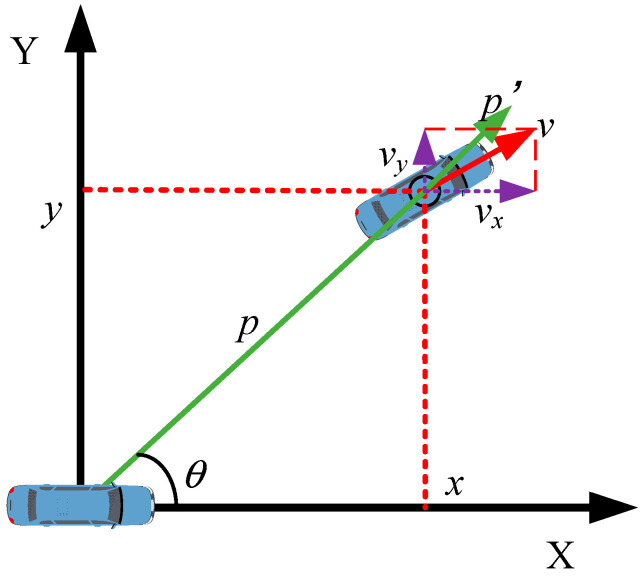
Coordinate transformation relationship.

The coordinate transformation relationship between the two sensors is illustrated in Figure 2.

### 3.2. SHAFEKF-Based Multi-Source Information Fusion

During the operation of intelligent vehicles, radar measurements demonstrate high precision. However, the presence of white noise and the accumulation of errors over prolonged use can result in decreased accuracy. Conversely, visual sensors deliver real-time state information about the vehicle. Consequently, an information fusion approach is employed to leverage the strengths of each sensor while mitigating their weaknesses, thereby yielding more accurate vehicle positioning data. In this context, the real-time measurement data from the radar are considered the predicted value, while image data from the camera are treated as the observed value for fusion purposes. The specific principle is outlined as follows.

The nonlinear system equation is represented as follows:

(6) $$\left\{\begin{array}{l} X_{k}=AX_{k-1}+\omega_{k-1}\\ Z_{k}=HX_{k}+v_{k} \end{array}\right.$$

where *A* is the state transition matrix, ω is the noise error, *H* is the measurement matrix, and *v_k_* is the measurement noise error.

The prior state equation is represented as follows:

(7) $$X_{k/k-1}=A_{k/k-1}X_{k-1}$$

where $X_{k}$ is the state estimation matrix and $A_{k/k-1}$ is the Jacobian of the state estimation matrix.

The error covariance prediction equation is represented as follows:

(8) $$P_{k/k-1}=A_{k/k-1}P_{k-1/k-1}A_{k/k-1}^{T}+B_{k-1}Q_{k-1}B_{k-1}^{T}$$

where $P_{k}$ is the error covariance matrix, $B_{k-1}$ is the Jacobian of the input noise matrix, and $Q_{k-1}$ is the system noise covariance matrix.

The residual equation is represented as follows:

(9) $$\varepsilon_{k}=Z_{k}-H_{k}X_{k/k-1}-r_{k}$$

where $H_{k}$ is the Jacobian matrix of the distance measurement function at $X_{k}$, and $r_{k}$ is the measurement noise variance.

The measurement update equation is represented as follows:

(10) $$K_{k}=P_{k/k-1}H_{k}^{T}{\left(H_{k}P_{k/k-1}H_{k}^{T}+R_{k}\right)}^{-1}$$

(11) $$X_{k}=X_{k/k-1}+K_{k}\varepsilon_{k}$$

(12) $$P_{k}=(I-K_{k}H_{k})P_{k/k-1}$$

where $K_{k}$ is the Kalman gain and $R_{k}$ is the measurement noise covariance matrix.

The adaptive estimator equation is represented as follows:

(13) $$r_{k}=(1-d_{k})r_{k-1}+d_{k}(Z_{k}-H_{k}X_{k/k-1})$$

(14) $${\hat{R}}_{k}=(1-d_{k}){\hat{R}}_{k-1}+d_{k-1}\varepsilon_{k}\varepsilon_{k}^{T}$$

(15) $$d_{k}=\frac{1-b}{1-b^{k+1}}$$

where *b* is the forgetting factor, which generally ranges from 0.95 to 0.995.

In multi-sensor fusion systems, complex environmental factors pose challenges in accurately determining the noise covariance matrix, which may change over time and thereby reduce fusion accuracy. Consequently, a gradually fading factor is introduced to adjust the predicted error covariance matrix, aiming to reduce estimation errors and mitigate the risk of filter divergence. As demonstrated in Equation (16),

(16) $$P_{k/k-1}=\lambda_{k}A_{k/k-1}P_{k-1/k-1}A_{k/k-1}^{T}+B_{k-1}Q_{k-1}B_{k-1}^{T}$$

From Equation (9), the theoretical value of the innovation’s covariance can be expressed as follows:

(17) $$C_{k}=E[\varepsilon_{k}\varepsilon_{k}^{T}]=H_{k}P_{k/k-1}H_{k}^{T}+{\hat{R}}_{k}$$

In the equation, *C_K_* exhibits a positive correlation with both $P_{k}$ and ${\hat{R}}_{k}$. The estimated value of *C_K_* obtained by the windowing method can be expressed as follows:

(18) $${\hat{C}}_{k}=\frac{1}{k-1}\sum_{i=0}^{k}\varepsilon_{i}\varepsilon_{i}^{T}$$

From Equations (9)–(18), the fading factor can be expressed as follows:

(19) $$\left\{\begin{array}{l} N_{k}={\hat{C}}_{k}-H_{k}B_{k-1}{\hat{Q}}_{k-1}B_{k-1}^{T}H_{k}^{T}-{\hat{R}}_{k}\\ M_{k}=H_{k}A_{k-1}P_{k-1}A_{k-1}^{T}H_{k}^{T}\\ \lambda_{k}=\left\{\max(1,tr(N_{k})/tr(M_{k}))\right\} \end{array}\right.$$

where *tr*() denotes the trace operation applied to a matrix. When the theoretical value of the innovation covariance diverges from the estimated value, adjustments to $\lambda_{k}$ are made to mitigate the growing estimation error. However, the value of ${\hat{C}}_{k}$ is calculated using the arithmetic average of the data within the sliding window, without incorporating the new incoming data. Thus, it is essential to design an exponentially weighted estimator with a gradually fading memory that prioritizes new measurement data, thereby emphasizing its importance. This paper assigns values to the weighting coefficients through a negative exponential function.

(20) $$\beta_{i-1}=\beta_{i}b,\;0<b<1,\sum_{i=1}^{k}\beta_{i}=1$$

(21) $$\left\{\begin{array}{l} b^{0}+b^{1}+b^{2}+b^{3}+\cdots +b^{k-1}=\frac{1-b^{k}}{1-b}\\ (b^{0}+b^{1}+b^{2}+b^{3}+\cdots +b^{k-1})\frac{1-b}{1-b^{k}}=1 \end{array}\right.$$

(22) $$m_{k}=\frac{1-b}{1-b^{k}}$$

(23) $$\beta_{i}=mb^{k-i},i=1,2,\cdots k$$

Substituting Equation (23) into Equation (18) yields the following results.

(24) $${\hat{C}}_{k}=\sum_{i=1}^{k}m_{k}b^{k-i}\varepsilon_{i}\varepsilon_{i}^{T}=m_{k}\varepsilon_{i}\varepsilon_{i}^{T}+\frac{m_{k}b}{m_{k-1}}m_{k-1}\sum_{i=1}^{k-1}b^{k-1-i}\varepsilon_{i}\varepsilon_{i}^{T}$$

(25) $$\frac{m_{k}b}{m_{k-1}}=\frac{\frac{1-b}{1-b^{k}}b}{\frac{1-b}{1-b^{k-1}}}=\frac{b-b^{k}}{1-b^{k}}=1-\frac{1-b}{1-b^{k}}=1-m_{k}$$

${\hat{C}}_{k}$ is presented as follows:

(26) $${\hat{C}}_{k}=m_{k}\varepsilon_{i}\varepsilon_{i}^{T}+(1-m_{k})m_{k-1}\sum_{i=1}^{k-1}b^{k-1-i}\varepsilon_{i}\varepsilon_{i}^{T}$$

The SHAFEKF algorithm presented in this article is detailed above.

The EKF necessitates iterative computation of the Jacobian matrix at each timestep along with matrix inversion operations, resulting in O(n^3^) computational complexity that escalates significantly for high-dimensional systems. While SHAEKF mitigates this burden through fixed sliding-window optimization (reducing complexity to O(kn^2^) where k is window size), our proposed SHAFEKF achieves superior efficiency by adaptively tuning window parameters using weight decay coefficients and implementing selective matrix recomputation protocols.

## 4. Experimental Results

**Figure 3:**
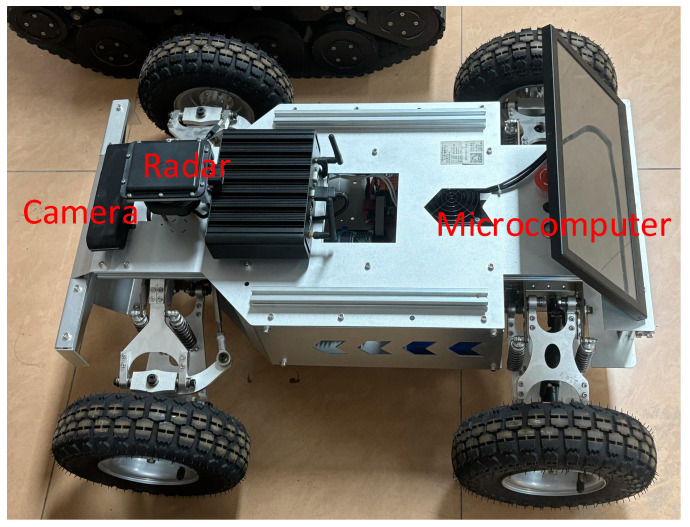
Mobile robot.

The method proposed in this paper is validated through a mobile robot that simulates an intelligent vehicle. This mobile robot is based on Ackermann steering geometry, which exhibits good maneuverability and performs well in indoor environments, as shown in Figure 3.

In addition to the connecting hardware, the robot is composed of a microcomputer, motors, servos, and sensors. The microcomputer is used to collect and process sensor data. The following microcomputer setup was used during the experimental: ubuntu 20.4, ARM Cortex-A72 CPU, Jetson Nano 4 GB GPU. The sensors include a camera and radar. The camera is a stereo depth camera capable of achieving a frame rate of 100 FPS, with a depth range of 0.3 to 15 m. The frequency band of the millimeter-wave radar is 77 GHz, with a maximum detection range of up to 300 m. The experiments were conducted in a laboratory environment, where one mobile robot acted as the host vehicle for obstacle detection, while another mobile robot served as a dynamic obstacle. This paper employs the Euclidean distance between two points on a two-dimensional plane as a measure of positional error.

**Figure 4:**
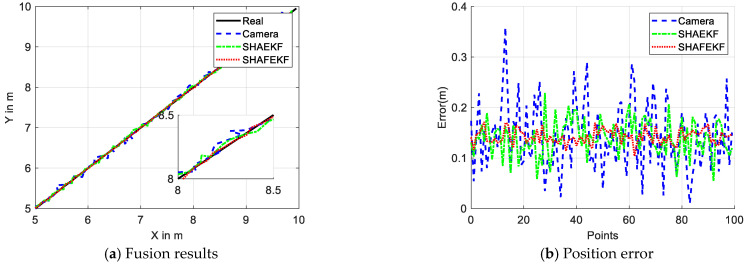
Output of the linear motion.

When the host vehicle is stationary, the dynamic obstacle moves in a straight line from 5 m to 10 m, as illustrated in Figure 4. Figure 4a indicates that the relative positioning error of the raw data captured by the camera is substantial, whereas the data fused using the SHAFEKF or SHAEKF algorithms exhibit a reduced relative position error. Moreover, Figure 4b reveals that the maximum detection error of the camera is 0.35 m. The SHAEKF algorithm has a maximum error of 0.22 m, while the SHAFEKF algorithm achieves a maximum error of 0.18 m, demonstrating the least fluctuation in error.

**Figure 5:**
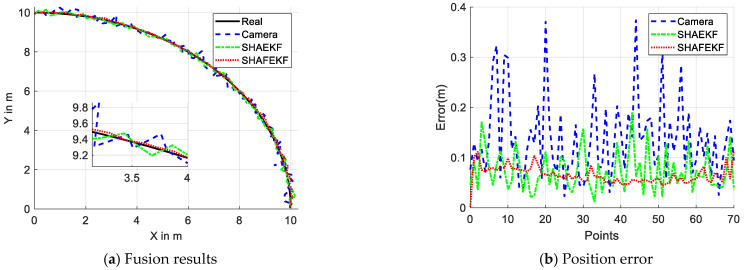
Output of the curvilinear motion.

When the host vehicle is stationary, the dynamic obstacle moves at a constant speed along an arc at a distance of 10 m, as illustrated in Figure 5. Figure 5a indicates that the relative position error of the data fused using the proposed SHAFEKF algorithm is relatively small. Additionally, Figure 5b reveals that the maximum detection error of the camera is 0.39 m. The maximum error of the SHAEKF algorithm is 0.19 m, whereas the SHAFEKF algorithm achieves a maximum error of 0.11 m.

To better validate the feasibility of the proposed algorithm, ten repeated experiments were conducted under identical experimental conditions. Table 1 presents the average error (AE) and mean squared error (MSE) for the camera, SHAEKF algorithm, and SHAFEKF algorithm. The data indicate that both the SHAEKF and SHAFEKF algorithms significantly improve positional accuracy while simultaneously reducing data volatility. Furthermore, the average error for the SHAFEKF algorithm decreased by 2.8% and 13.4% in comparison to the SHAEKF algorithm across the two scenarios, while the mean squared error decreased by 64% and 72%. These findings demonstrate that the SHAFEKF algorithm offers advantages of enhanced accuracy and reduced fluctuation, thus providing greater adaptability in multi-source information fusion systems.

## 5. Discussion

With the advancement of intelligent driving technologies, multi-sensor fusion has emerged as the predominant development direction for next-generation environmental perception systems. By synergistically leveraging cameras’ high-resolution image processing capabilities and millimeter-wave radars’ precise velocity measurement characteristics, this approach achieves robust object detection under adverse conditions including low-light scenarios and inclement weather (e.g., rain/fog). It effectively compensates for inherent limitations of individual sensors in extreme weather or complex occlusion scenarios, such as optical interference affecting cameras and angular resolution constraints in radar systems.

The proposed SHAFEKF algorithm significantly enhances obstacle detection accuracy for intelligent electric vehicles during operation. This improvement enables the driving system to make faster decisions during emergency responses in complex traffic environments. Furthermore, the precision of obstacle detection directly impacts vehicular navigation and driving decisions, where high-accuracy detection better supports critical functions including path planning and dynamic obstacle avoidance. Through its fading memory exponential weighting filter estimator, the SHAFEKF algorithm enhances the utilization weight of newly acquired data. Although we have not yet tested under conditions such as rain, fog, or electromagnetic noise, we have observed that the sensors inherently introduce measurement noise. Our results indicate that SHAFEKF achieves a 15% improvement in measurement accuracy compared to traditional camera systems, demonstrating its adaptability to sensor degradation. This innovation achieves more reliable perception under adverse weather conditions with increased measurement noise (e.g., rain and fog), ensuring safe vehicle operation across diverse scenarios. The algorithm’s adaptive capability proves essential for realizing comprehensive intelligent driving, particularly in maintaining robust environmental awareness during sudden sensor degradation or transient interference events. In our future research, we will concentrate on exploring real vehicle scenarios across diverse environments to further validate our findings.

## 6. Conclusions

Multi-source information fusion technology in intelligent driving perception systems has evolved into a highly interdisciplinary and integrated research field. This technology enhances the accuracy and robustness of environmental perception systems. This paper investigates a multi-source information fusion system in which the data from one sensor serve as a predictive value, while the data from another sensor act as a measurement value for the fusion process. We introduce an improved SHAEKF algorithm by incorporating a fading factor that reduces estimation errors and mitigates the risk of filter divergence. Experimental results demonstrate that this algorithm achieves high estimation accuracy within the multi-source information fusion system. However, the system examined in this study does not account for the influence of diverse sensor types and varying environmental factors. Expanding the diversity of sensors under different environmental conditions is a critical consideration for future research.

## Figures and Tables

**Table 1:** Performance comparison of fusion systems.

	Camera		SHAEKF		SHAFEKF	
	AE	MSE	AE	MSE	AE	MSE
linear	0.163	4.9 × 10^−3^	0.141	1.3 × 10^−3^	0.137	4.6 × 10^−4^
curvilinear	0.145	7.7 × 10^−3^	0.082	1.4 × 10^−3^	0.071	3.9 × 10^−4^

## Data Availability

The data supporting this study are available from the corresponding author upon reasonable request.
